# Streamlining recombinant peptide and protein production using a pre-configured elastin-like polypeptide expression cassette

**DOI:** 10.1371/journal.pone.0357387

**Published:** 2026-09-11

**Authors:** Jetson Lobos, Jeffrey Velasquez, Fariborz Nasertorabi

**Affiliations:** 1 Bridge Institute, Michelson Center for Convergent Bioscience, Structure Biology Center, University of Southern California, Los Angeles, California, United States of America; 2 Bridge Institute, Michelson Center for Convergent Bioscience, University of Southern California, Los Angeles, California, United States of America; Arizona State University, UNITED STATES OF AMERICA

## Abstract

Elastin-like polypeptides (ELPs) are synthetic biopolymers that exhibit a unique thermo-responsive behavior: they remain soluble below a defined transition temperature but undergo reversible aggregation when the temperature exceeds this threshold. This property enables a simplified, non-chromatographic protein purification strategy. Despite these advantages, ELP-based purification has not yet been widely adopted as a standard method in research laboratories, indicating that certain practical limitations or challenges remain to be addressed. The first major challenge is the absence of a standardized ELP-tagged protein expression cassette suitable for use in research laboratories. The second is the development of an effective strategy for purifying the target protein released from the ELP tag following enzymatic cleavage. In this study, we present an optimized and modular gene cassette designed to standardize ELP-tagged peptide that can operates under mild conditions, featuring a low inverse transition temperature (Tt) of 27–31 °C, reduced salt concentrations (up to 0.5 M NaCl), and produces high yield and high purity product. Additionally, we exploited the ELP’s concentration induced aggregation to facilitate its separation from the target protein by filtration following enzymatic cleavage to address the second challenge. Here, we emphasize that this protocol has proven to be particularly efficient for the purification of small peptides, primarily due to the substantial size difference between the target peptide and the ELP tag. The purification of larger peptides remains feasible but may require alternative purification strategies. As a case study, we illustrate the purification of human β-defensin 1 (hBD-1; gene *DEFB1*), a 36-residue antimicrobial peptide, and discuss the challenges encountered and solutions implemented during the purification process.

## Introduction

The expression and purification of proteins remain significant challenges in research laboratories. To achieve soluble expression of target proteins, researchers commonly employ strategies such as N- and/or C-terminal truncations, site-directed mutagenesis, selection of appropriate fusion partners, and choice of expression systems.

Once soluble expression is confirmed, a combination of purification techniques is typically employed, guided by factors such as protein size, net charge, and the nature of any fused affinity tags. The combination of three step purification is time consuming, expensive, requires expensive chromatography system.

The discovery of elastin-like polypeptides (ELPs) and their reversible aggregation properties has unlocked significant potential for their application in medical and biomedical fields, including drug delivery [1,2] tissue engineering [3] and protein purification [4,5]. ELPs are considered a safe option due to their biological compatibility, non-pyrogenic nature, and lack of immunogenicity [6,7].

ELPs are synthetic biopolymers that mimic tropoelastin, the human precursor to elastin. They consist of repetitive sequences of five amino acids (VPGXaaG), where Xaa can be any amino acid except proline [8]. ELPs exhibit highly disordered structures, primarily due to their low-complexity amino acid sequences, which typically consist of over 80% hydrophobic residues—namely valine, proline, and glycine. This compositional bias contributes to their intrinsic disorder and conformational flexibility in solution [9,10]. These biopolymers exhibit a unique thermo-responsive behavior, remaining soluble below a specific transition temperature and forming reversible aggregates when the temperature exceeds this threshold. The process in which proteins are aggregated by raising the temperature above their transition temperature and subsequently pelleted by centrifugation (“hot spin”) and then resolubilized by lowering the temperature below the transition temperature, followed by a second centrifugation step (“cold spin”) to remove insoluble impurities is called by the term Inverse Transition Cycling (ITC) [11].

The transition temperature is primarily determined by the identity of the residue Xaa within the repeating sequence, although factors such as the length of the sequence, protein concentration, salt concentration, and pH also influence the aggregation behavior [7,12–15]. There have been several studies aiming to accurately predict the transition temperature of elastin-like polypeptides by considering variables such as amino acid sequence, chain length, concentration, and environmental conditions [12,16,17]. These models facilitate the rational design of ELPs tailored to specific biomedical or biotechnological applications.

Theoretically, fusing elastin-like polypeptides to a protein of interest offers an attractive approach for one-step purification without the need for chromatography. Several successful applications of this technique have been reported, providing strong evidence for its efficacy and encouraging further exploration [5,18–20].

Despite the promising potential of this method, several challenges persist, which may discourage researchers from adopting it in their laboratories. One significant difficulty is the lack of a standardized ELP sequence suitable for the expression and purification of commonly studied biochemical and medically relevant proteins. As a result, researchers must use the prediction models or empirically select the appropriate ELP sequence, length, possible tags and enzyme cleavage site for each target. Additionally, we observed that small modifications to the C- or N-terminal regions of the ELP sequence can drastically impair protein expression. This prompted us to search for a standardized ELP sequence that could facilitate the cloning and protein production through simple insertion of the target protein, followed by testing for expression and solubility.

In this work, we introduce a construct specifically designed to facilitate the efficient production of ELP-tagged peptides and proteins in research laboratory settings. The system allows for the straightforward insertion of a target gene to rapidly assess both expression and solubility in a single step. The construct incorporates a predefined ELP sequence (ELP[V-96]), a flexible linker, and a TEV protease cleavage site which leaves a serine at the N-terminal of the target protein after its release by enzymatic cleavage. Importantly, the design is modular, enabling users to easily modify the arrangement at either terminus to suit specific experimental needs.

Additionally, we leveraged the intrinsically disordered nature of ELP as a means to separate it from the target protein following enzymatic cleavage, using a simple filtration-based approach. ELP’s lack of defined tertiary structure stems from its repetitive pentapeptide sequence, which is rich in hydrophobic residues. ELP tends to aggregate at high concentration and therefore impedes its ability to pass through centrifugal filters designed for globular proteins of similar molecular weight. As a result, the released, structured target protein can efficiently pass through the membrane, while the unstructured ELP is retained—enabling effective separation without the need for chromatographic methods.

The protocol has been demonstrated to be effective for the production and purification of small proteins and peptides, primarily due to the substantial size difference between the target protein and the ELP tag. However, purification of larger proteins and peptides may require alternative strategies, such as incorporating an affinity tag into the target protein or developing more innovative approaches to achieve efficient separation of the target protein from the ELP tag following cleavage.

As a case study to demonstrate the functionality and efficiency of our ELP-based expression system, we carried out the cloning, expression, and purification of human β-defensin 1 (hBD-1; gene *DEFB1*), a well-characterized antimicrobial peptide consisting of 36 amino acid residues. hBD-1 plays a critical role in innate immunity and is of significant interest in both biomedical and biochemical research [21,22]. Recombinant production of this peptide presents a notable challenge because of its smaller size, being positively charged and therefore prone to degradation. This example highlights the practical utility of our system as a streamlined approach for purifying large quantities of peptides or proteins at high purity, without the need for chromatographic techniques. A step-by-step protocol is provided as supporting material (S1 Protocol).

## Materials and Methods

### Construct design

**Fig 1:**
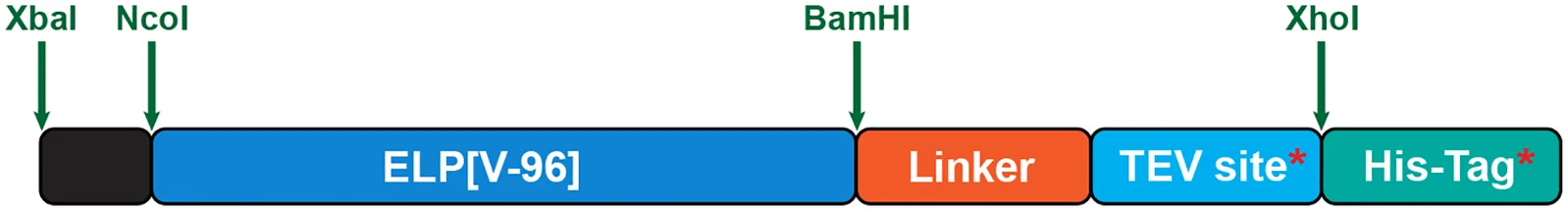
Strategic incorporation of restriction enzyme recognition sites facilitates an efficient and easy way for genetic manipulation and customization of the cassette. XbaI/NcoI or NcoI sites: To place any desired sequence like tag, cleavage site, linker and/or target protein at the N-terminal of the ELP sequence. NcoI/BamHI sites: To allow users to move the ELP sequence to other plasmids or replace the current ELP sequence with any other ELP sequences to change its characteristics. BamHI/XhoI: A Linker and a TEV cleavage sequence is already placed at the C-terminal of the ELP sequence between these two restriction sites; however, one could use these sites to introduce a new linker, cleavage site or an affinity tag. Stop codon: A stop codon has been placed just before the XhoI restriction site. By eliminating the stop codon, a sequence containing a 6xHis-tag (LEHHHHHH) will automatically be added C-terminally to the target protein. A red star (*) indicates a stop codon.

The original ELP construct was generously provided by Dr. J. Andrew MacKay from the USC School of Pharmacy. This construct consisted of 96 tandem repeats of the pentapeptide sequence VPGVG and it has shown to have a Tt of 31.9°C at 25uM [6]. While this construct exhibited robust ELP expression, its design was not ideally suited to the needs of our experimental workflow. Consequently, we developed a modified version that facilitates easier genetic manipulation and customization. In this study, we employed the pET-28 vector as the backbone for all constructs. Both ELP and the target sequences were codon-optimized for *Escherichia coli* expression and synthesized by Genscript (NJ, USA). Cloning of the target genes into the expression vector was performed either by Genscript or in-house. All constructs were validated by Sanger sequencing to confirm sequence accuracy. Due to the repetitive nature of the ELP sequence, conventional primer-based methods for genetic modification are impractical. To overcome this limitation, we have strategically incorporated restriction enzyme recognition sites within the gene, enabling modular assembly of gene cassettes (Fig 1).

### Control constructs expression

Three protein constructs including two constructs from Nuclear Factor of Activated T cells (NFAT) and one from Glutamine--fructose-6-phosphate aminotransferase (GFAT) which had been previously tested for expression with a His-tag as an affinity tag were cloned into the ELP construct cassette for comparative analysis. All target sequences were codon-optimized for bacterial expression and synthesized by Genscript (NJ, USA).

### Expression and initial screening of recombinant protein

A single colony of *E. coli* harboring the expression plasmid encoding the target protein was inoculated into 2 mL of Luria Broth (LB) supplemented with 50 μg/mL kanamycin. The culture was incubated at 37 °C with shaking at 200 rpm until it reached visible turbidity, indicating robust cell growth. At this point, the incubation temperature was reduced to 18 °C. Protein expression was induced by adding isopropyl-β-D-thiogalactopyranoside (IPTG) to a final concentration of 0.25 mM, and the culture was incubated overnight at 18 °C with shaking at 220 rpm. The following day, cells were harvested by centrifugation at 6,000 × *g* for 15 minutes at 4 °C. The supernatant was discarded, and the cell pellet was resuspended in 300 μL of lysis buffer (50 mM HEPES, pH 7.5; 200 mM NaCl; 1 mM AEBSF). Cell lysis was performed using a cold-water bath sonicator until the lysate became less viscous and more transparent.

The lysate was then centrifuged at 23,000 × *g* for 10 minutes at 4 °C to remove cell debris. The resulting supernatant was transferred to a 30 °C water bath and incubated for 15 minutes. A visible increase in turbidity of the supernatant indicated successful expression of the recombinant protein, suggesting suitability to scale-up.

### The protocol for expression and purification of hBD-1 peptide

The protocol described in this peer-reviewed article is published on protocols.io (DOI: https://dx.doi.org/10.17504/protocols.io.bp2l6ob9klqe/v1) and is included for printing purposes as S1 Protocol.

The complete sequence of the vector harboring the expression cassette for ELP[V96]-hBD-1 is included in the supporting Information (S1 File Vector sequence ELP[V96]-hBD-1 pET-28). The vector can be obtained upon request.

## Results and discussion

The ELP[V96] cassette enables users to tailor the expression construct by incorporating desired tags, linkers, and enzymatic cleavage sites. The cassette was designed to support a straightforward one-step cloning strategy for generating expression plasmids in standard research laboratories. Based on our experience, placing the target protein immediately downstream of the TEV cleavage site results in improved expression and purification efficiency.

### Purification of the fused protein

#### 1^st^ round of ITC.

**Fig 2:**
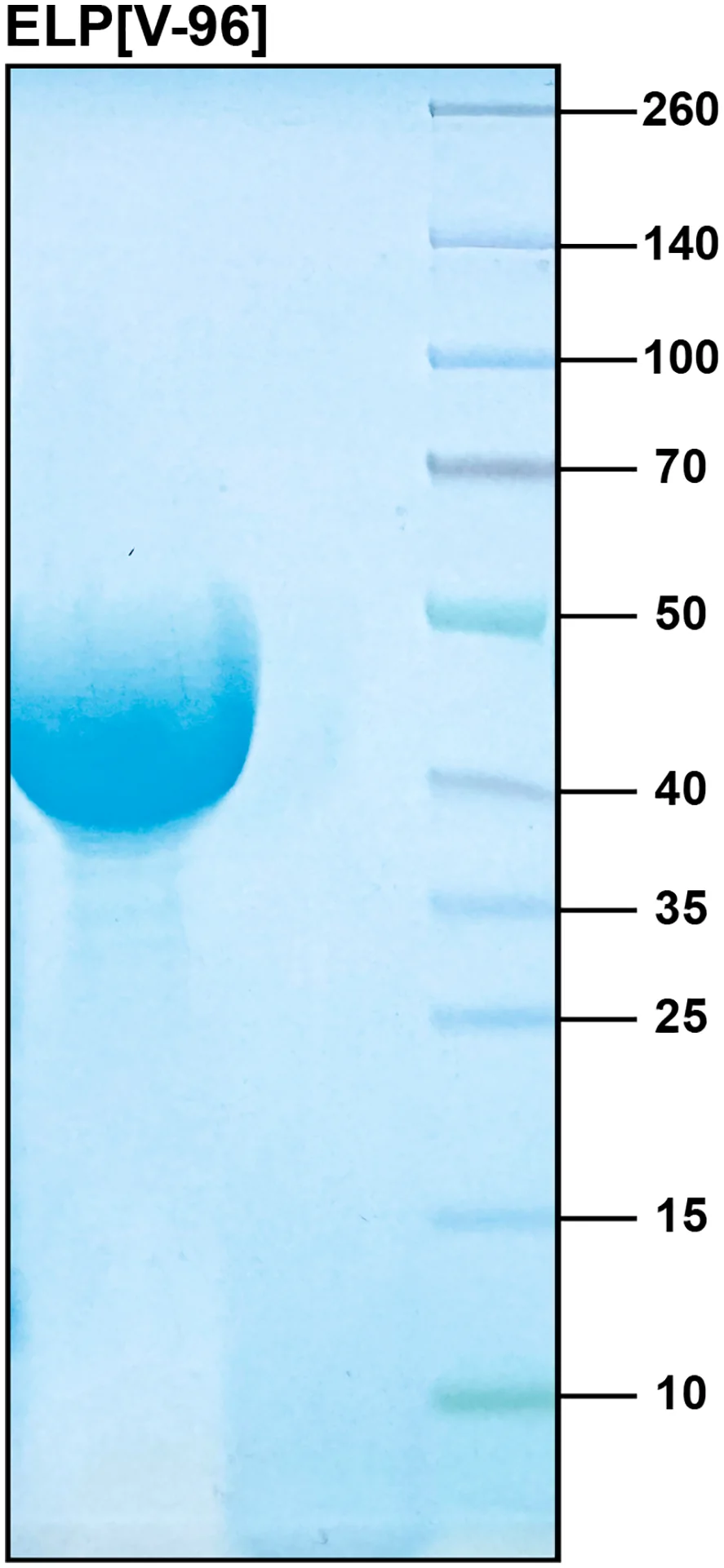
One round of ITC for ELP [V96] yields high protein purity. 10 ul of the dissolved pellet from the hot spin has been loaded onto a 4–12% gradient SDS PAGE.

A single round of inverse transition cycling (hot spin/cold spin) typically yielded a fusion protein purity of over 85%. However, the final purity can vary depending on the nature of the inserted sequence. For instance, the ELP construct ELP[V-96] without any fusion insert consistently achieved a purity level exceeding 95% (Fig 2).

#### Hot Spin.

The transition temperature of the ELP[V-96] construct was observed within the range of 27–31 °C in the presence of 200 mM NaCl, representing the onset and completion of aggregation. This temperature can be fine-tuned for individual target proteins by adjusting salt concentration; for example, a lower Tt can be achieved by increasing the ionic strength of the solution. In our protocol, we selected a Tt of 31 °C, as the target protein demonstrated stability under these conditions and exhibited no signs of proteolytic degradation. This stability is attributed to the protective effect of the large ELP[V-96] fusion, which shields the charged peptide from protease activity. For larger proteins, it is generally advisable to maintain a lower transition temperature and compensate by increasing the salt concentration to promote efficient aggregation. This approach helps preserve protein stability while maximizing recovery during the phase transition process.

#### Cold spin.

The sample was centrifuged to remove impurities and any undissolved proteins. The pellet was discarded, and the supernatant was subsequently filtered through a 0.45 μm membrane filter to eliminate remaining large particulates. If cleavage of the ELP tag is not required for the intended application, the purified fusion protein typically attains a purity level of 80–90%. However, when removal of the ELP tag is necessary, additional purification steps are required.

### 2^nd^ round of ITC

If the purity of the preparation is insufficient, an additional round of inverse transition cycling may be performed. In our experience, the second round of hot/cold cycling provided only marginal improvement in purity and was generally avoided to minimize sample processing time and potential protein loss.

The transition temperature typically decreased by several degrees after cellular proteins were removed during the initial purification step. For example, in the case of hBD-1, the transition temperature shifted to approximately 24–27 °C.

### Micro-filtration

There is a report describing successful purification of ELP fusion proteins using microfiltration following heat treatment [23]. In theory, the aggregated protein should be retained by the filter, allowing soluble proteins to pass through. Based on our experience, this approach can be effective for small-scale purifications, but it is prone to rapid filter clogging with larger volumes.

### Isolating the target protein released from ELP

Following cleavage of the target protein from the ELP tag, the solution contains a mixture of the liberated target protein, free ELP[V-96], TEV protease, and residual un-cleaved fusion protein. Various purification strategies can be employed to isolate the target protein, and the optimal approach may vary depending on the specific protein. Here, we briefly describe several methods used to isolate the target protein and discuss the specific challenges associated with each approach.

1- **Hot spin**

**Fig 3:**
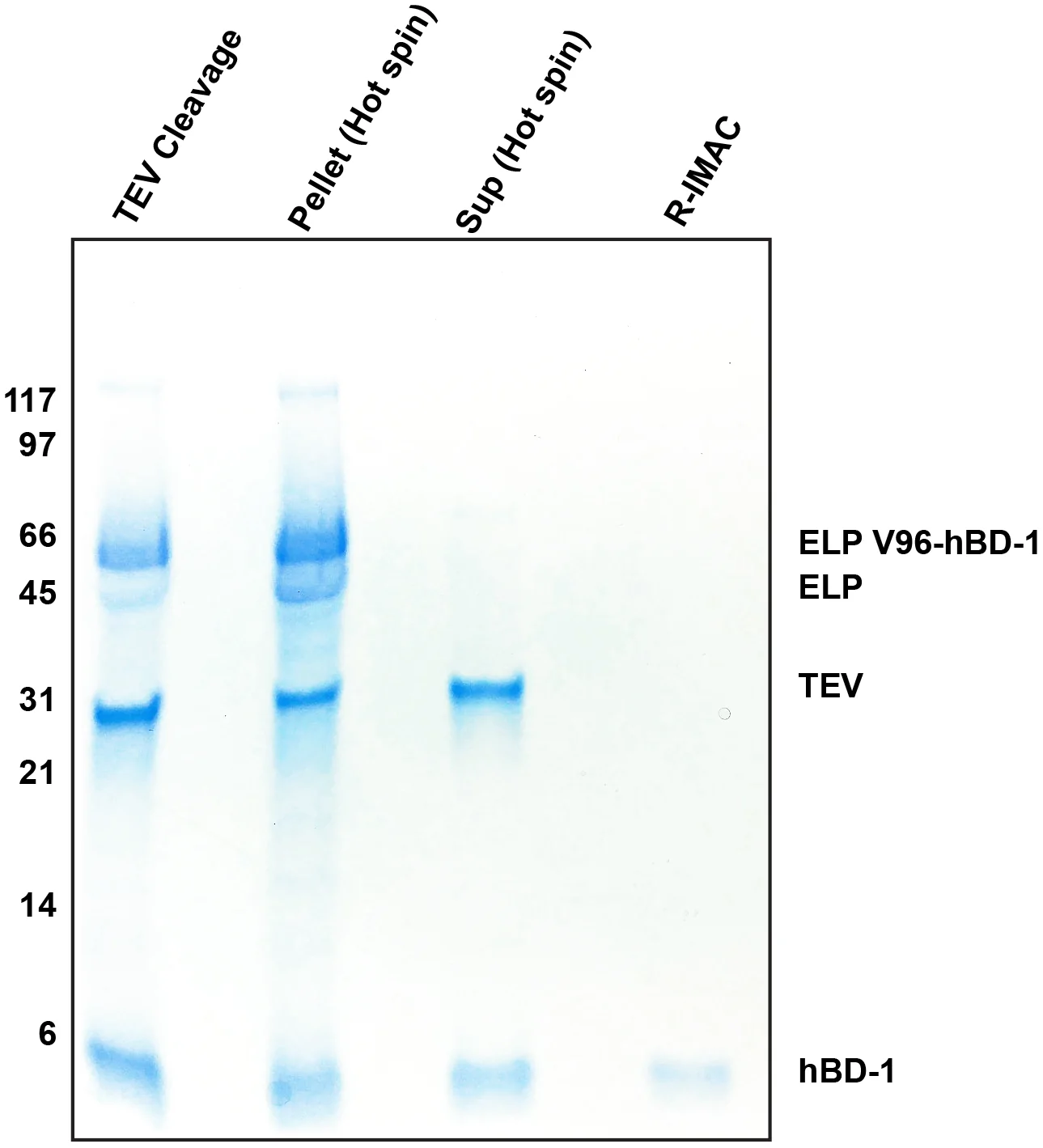
Soluble proteins co-aggregate with ELP during the hot spinspin. Both TEV and hBD-1 are found in the pellet during the hot spin indicating a partial co-aggregation of the soluble proteins with ELP.

While this method is generally effective, we observed that a significant fraction of both the target protein and TEV protease co-sediments with the ELP pellet during the heat-induced aggregation step (Fig 3). A several fold dilutions can help to minimize co-sedimentation, but it will subsequently increase the sample volume posing practical handling challenges.

2- **Ion Exchange Chromatography (IEX)**

The presented ELP sequence lacks intrinsic charged residues, except for two negatively charged amino acids located within the linker-TEV protease cleavage site module. Due to this minimal overall charge, the 41 kDa ELP construct exhibits limited interaction with charged chromatographic resins and typically flows through without significant retention. In contrast, the target protein if possessing a stronger net surface charge can interact with the charged matrix, allowing for selective binding and subsequent elution. This charge-based separation strategy should provide a practical approach for isolating the target protein from the cleaved ELP and residual fusion components, however, we noticed that the charge-based separation does not happen effectively at high protein concentration, therefor a several-fold dilution is recommended to improve the binding efficiency to achieve higher yield.

3- **Affinity Chromatography**

While the addition of an affinity tag should enable efficient isolation of the target protein, similar to ion exchange chromatography, it is advisable to dilute the sample several-fold in expense of diluting the sample several folds.

4- **Micro Filtration**

Microfiltration is an efficient method for separation of the soluble proteins from the aggregated ELP; however, its application is limited to small-volume samples due to membrane clogging caused by aggregated ELP.

**5- Purification by filtration**

The cleaved sample was first processed using a 30 kDa MWCO centrifugal filter. ELP, being a large and unstructured molecule generates a viscous solution at high concentration, was effectively retained, leaving behind a dark brownish retentate that predominantly consisted of ELP and TEV. The filtrate, enriched with smaller components, was subsequently passed through a 10 kDa MWCO centrifugal filter to remove any residual ELP and TEV protease that may have leaked through the 30 kDa MWCO centrifugal filter.

The final filtrate was then concentrated using a 3 kDa MWCO centrifugal filter to enrich the hBD-1peptide. The purity and identity of the final product were verified by SDS-PAGE and mass spectrometry, confirming the successful isolation of hBD-1 at high purity. At the industrial scale, tangential flow filtration (TFF) can be an effective method for processing large volumes, enabling the efficient separation of small peptides such as hBD-1 from higher molecular weight contaminants.

At this stage a size exclusion chromatography can be utilized to assess the purity and oligomerization state of the final product.

### Size Exclusion chromatography for analytical purposes

**Fig 4:**
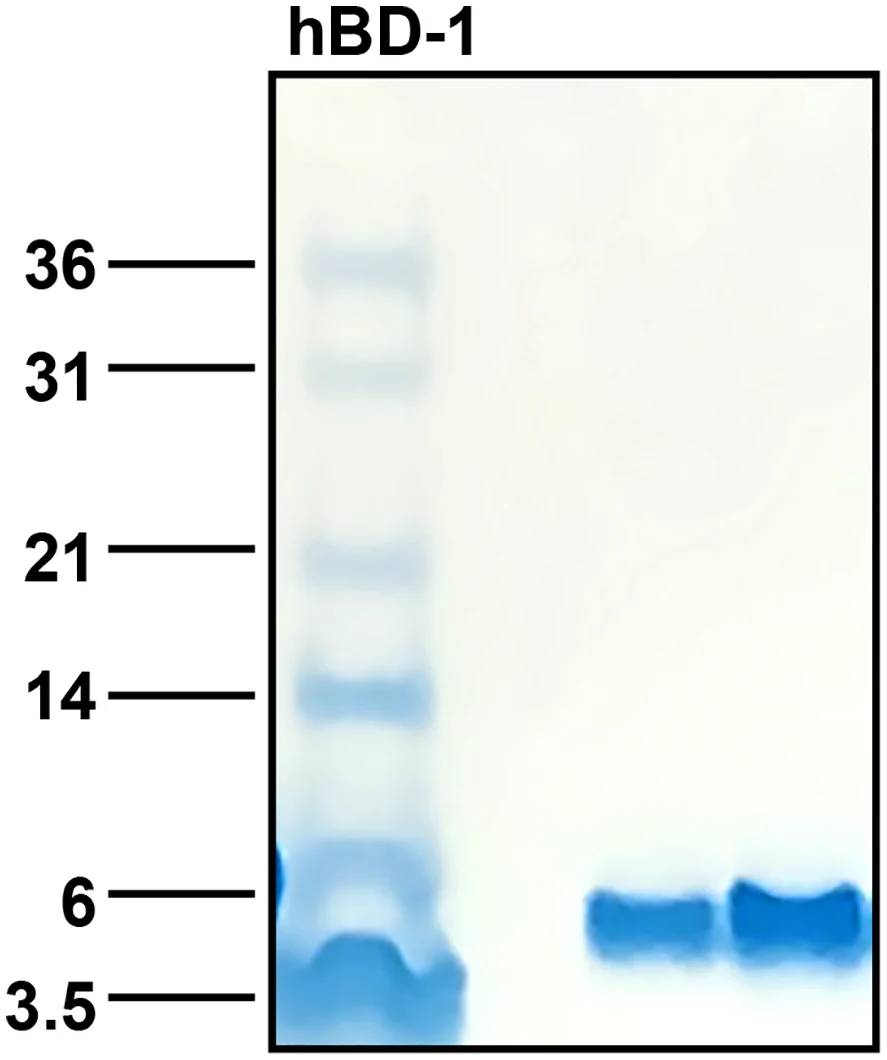
Purified hBD-1 was concentrated and ran on a Superdex 75 Increase 10/300 GL size exclusion column to confirm its purity and multimerization status. 20 ul of fractions from Size Exclusion Column has been loaded onto a 4–12% gradient SDS PAGE.

To examine the multimerization or aggregation status of purified hBD-1, the final sample was concentrated and loaded on a Superdex 75 Increase 10/300 GL size exclusion chromatography. hBD-1 was eluted with no evidence of aggregation or multimerization. SDS-PAGE analysis of the collected fractions confirmed its high purity (Fig 4).

If the peptide does not pass efficiently through a 10 kDa MWCO centrifugal filter, the 30 kDa MWCO filtrate can be concentrated and applied to a size-exclusion column, which will readily separate the small peptides from any remaining TEV and V96 in the solution.

### ELP vs His-tag

A total of three His-tagged constructs, previously evaluated for expression, were inserted into the ELP[V-96] construct immediately downstream of the TEV cleavage site and subsequently tested for expression. In addition, we successfully expressed and purified two small peptides, namely Stress-associated endoplasmic reticulum protein 1 (SERP1) and Human beta defensin-1 (hBD-1) fused to ELP, for which fusion to a His-tag was not practical or effective. Solubility test and expression of cells resulted in two soluble proteins from ELP compared to one soluble protein from His-tag expression system (Table 1).

**Table 1:** Two peptide sequences and three protein constructs were evaluated using the ELP cassette system.

Target	Uniprot	Range	Residues	MW (kDa)	Soluble expression	
8x-His	ELP
hBD-1	P60022	32-68	37	4.0		+
SERP1	Q9Y6X1	2-42	42	4.7		+
NFAT	Q13469	1-415	415	43.6	–	+
NFAT	Q13469	94-185	92	9.6	+	+
GFAT	Q06210-2	1-260	260	30.3	–	–

The protein constructs had previously been assessed for expression using an N-terminal His-tag for affinity purification. Both NFAT constructs and the two peptides were highly expressed using this ELP cassette.

**Fig 5:**
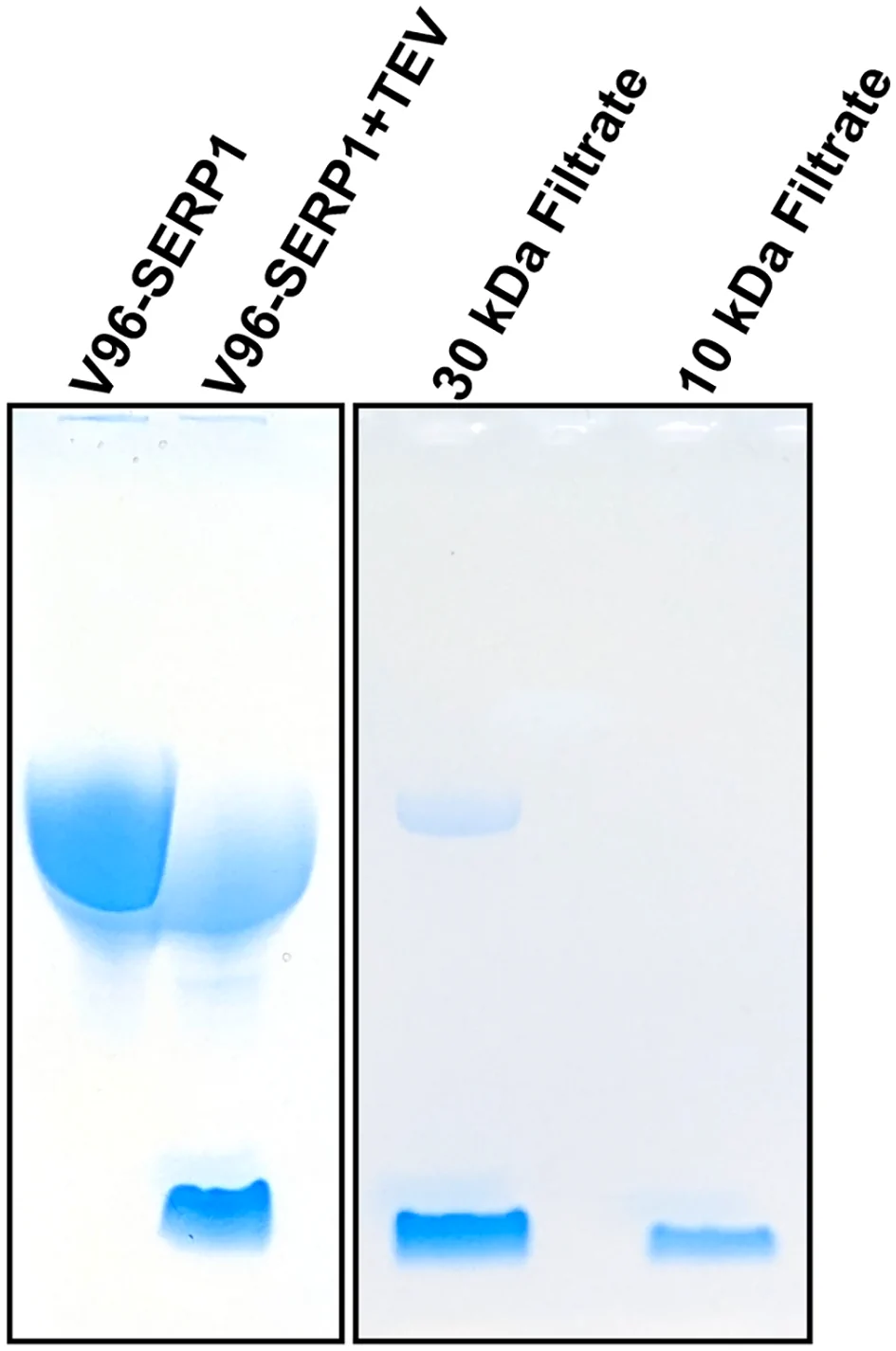
Purification of SERP1 peptide using ELP system with no chromatography. 20 ul of each purification step has been loaded onto a 4–12% gradient SDS PAGE.

Separation of the target protein from ELP following enzymatic cleavage proved less efficient when the molecular sizes of the released peptide and ELP were similar but was considerably more straightforward when their sizes differed. Apart from the hBD-1 peptide, we were able to purify large quantities of the Stress-associated endoplasmic reticulum protein 1 (SERP1) peptide using the same methodology, thereby demonstrating that ELP-based fusion is an efficient, practical, and straightforward approach for small peptide production (Fig 5). Production of peptides using affinity tags is neither practical nor efficient, but it works perfectly well using ELP method.

### Purification of larger proteins

**Fig 6:**
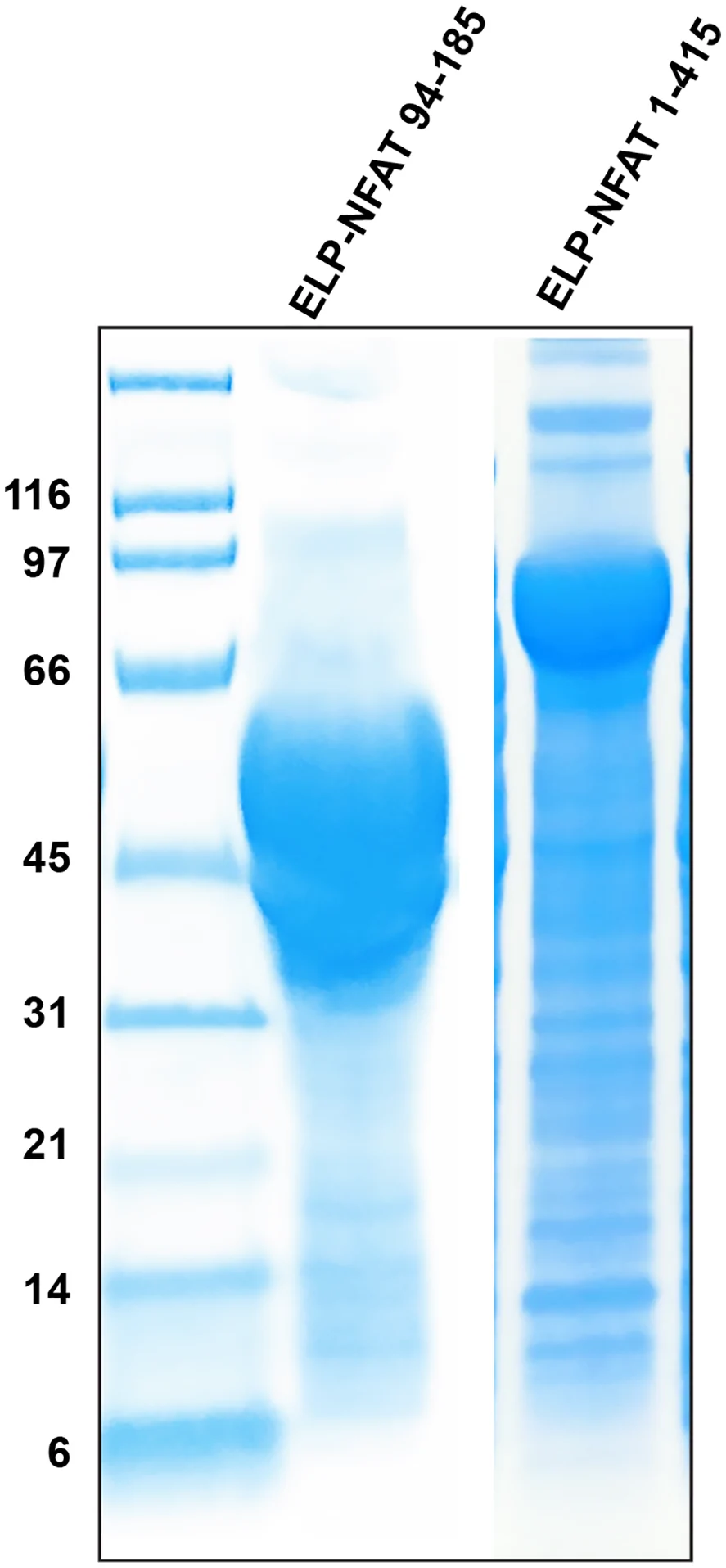
Both ELP-NFAT 94-185 and ELP- NFAT 1-415 were isolated at the first hot spin step. 20 ul of dissolved pellet from each sample was loaded onto a 4–12% gradient SDS PAGE.

Among the three proteins tested, two NFAT 94–185 and NFAT 1–415 were expressed at high levels and could be isolated in high yield during the initial hot-spin purification step (Fig 6).

**Fig 7:**
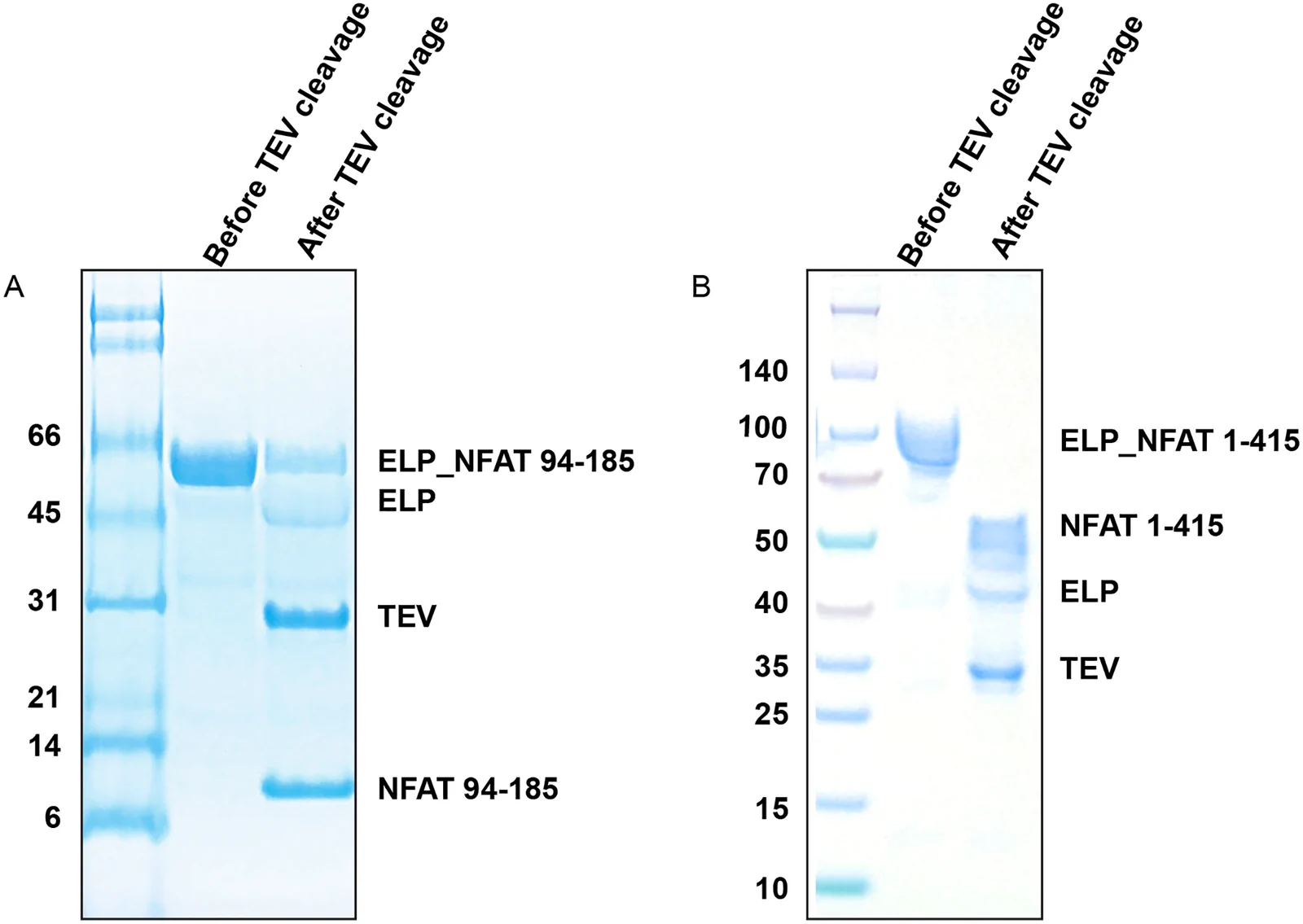
The enzymatic cleavage of both NFAT 94-185 and NFAT 1-415 releases the target proteins.

Both NFAT 94–185 and NFAT 1–415 were exposed to enzymatic cleavage by TEV after the first round of ITC (Fig 7). The samples were the subjected to an additional hot-spin step to remove residual ELP from the solution. ELP was discarded, and the soluble fraction containing the target proteins was retained for further purification.

Enzymatic cleavage of ELP constructs A) ELP-NFAT 94–185 cleaved with TEV, has released NFAT 94–185 from ELP. B) ELP-NFAT 1–415 cleaved with TEV, has released NFAT 1–415 from ELP.

**Fig 8:**
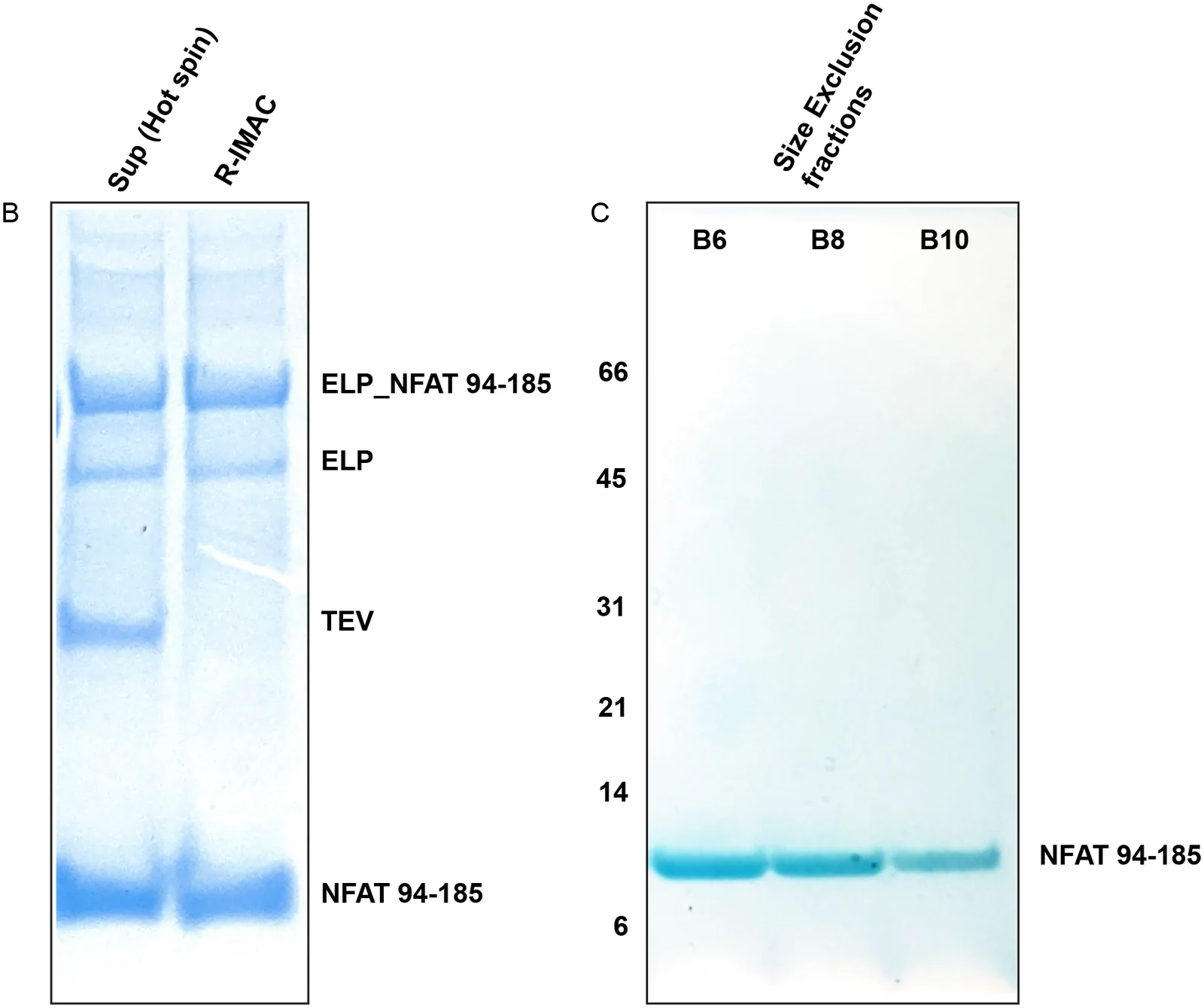
Purification of NFAT 94-185 after the TEV cleavage followed by a hot spin, R-IMAC and SEC. A) The soluble fraction after a hot spin. B) Flow through from a reverse IMAC. C) 0.5 mL elution fractions collected by size-exclusion chromatography (Superdex 200 increase 10/300 GL with a void volume of 10 mL) with the major peak eluting at a retention volume of 19.1 mL over an elution volume range of 18.4–20.5 mL.

NFAT 94–185 was subsequently subjected to a reverse immobilized metal affinity chromatography (IMAC) step to remove TEV, before it was concentrated and loaded onto a size-exclusion chromatography using Superdex 200 increase 10/300 column (Cytiva, USA), to remove remaining ELP (Fig 8).

**Fig 9:**
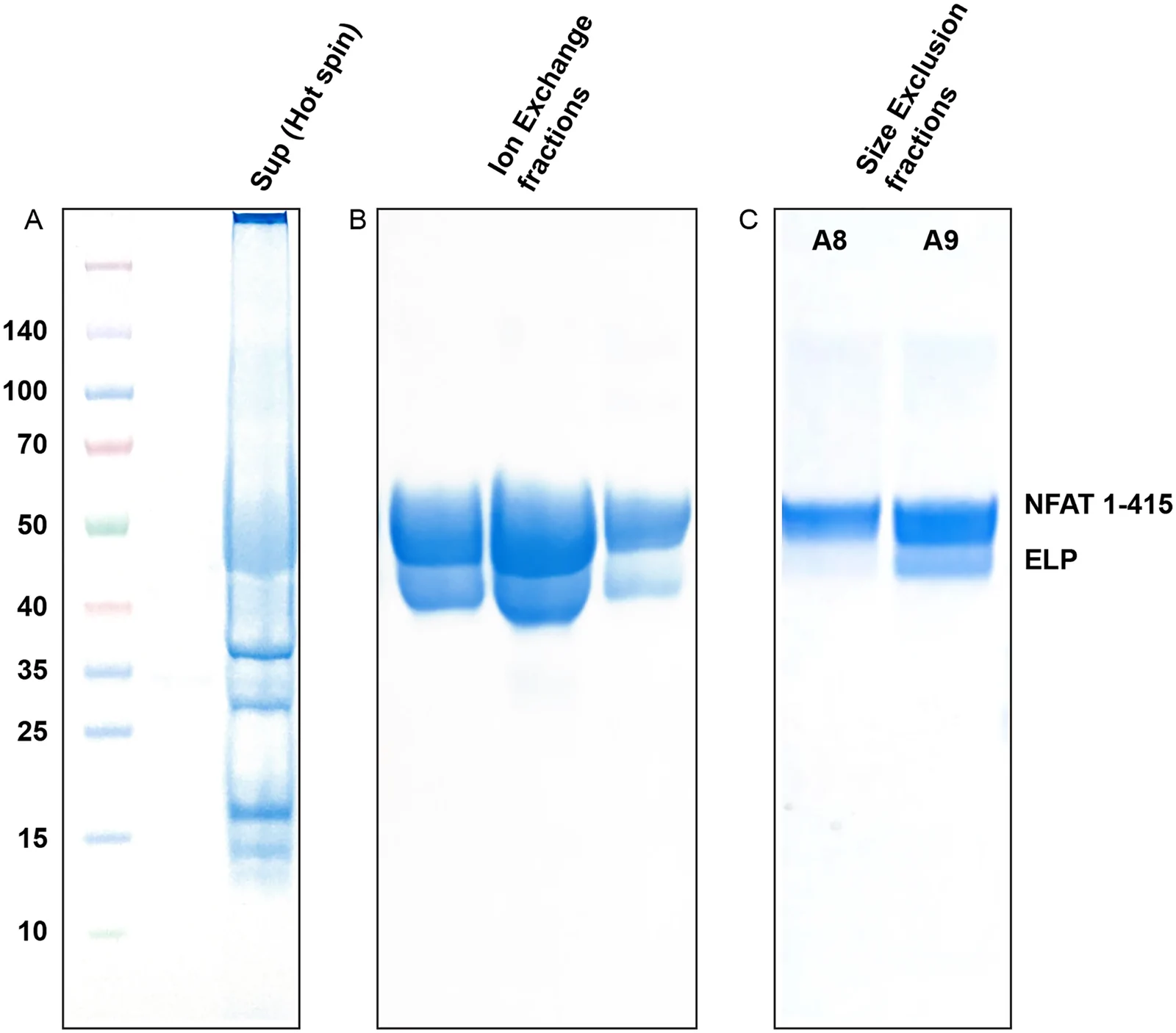
Purification of NFAT 1-415 after the TEV cleavage followed by a hot spin, Anion Exchange and SEC. A) The soluble fraction after a hot spin. B) Fractions from Anion Exchange elution. C) 0.5 mL fractions collected by size-exclusion chromatography (Superdex 200 increase 10/300 GL with a void volume of 10 mL) with the major peak eluting at a retention volume of 15.1 mL over an elution volume range of 14.2–16.7 mL.

In contrast, NFAT 1–415 was applied directly to an anion-exchange chromatography column and eluted using a salt gradient. The peak fractions were concentrated and loaded onto a size-exclusion chromatography using Superdex 200 increase 10/300 column (Cytiva, USA), to remove remaining ELP. However, this approach separate NFAT 1–415 from ELP partially (Fig 9).

## Conclusions

In this study, we present a robust and adaptable ELP-based expression construct suitable for routine laboratory use. This system enables efficient production of both peptides and proteins without subjecting the target molecules to extreme heat or high salt concentrations that could compromise stability or activity. The construct is modular by design, allowing for facile modifications to incorporate different ELP variants, protease cleavage sites, or affinity tags on either terminus. This versatility makes the system a practical and scalable solution for both basic research and applied protein production.

We identified a key advantage of the ELP system: its ability to support the efficient expression and purification of small peptides, which are often challenging to produce using conventional methods. Peptides offer several benefits that are particularly advantageous during both the production and purification processes. Due to their small size, peptides exert minimal influence on the expression efficiency and the inverse transition temperature of the ELP-fusion protein. The substantial size difference between the ELP tag and the peptide also enables the use of filtration-based methods in a more effective way, minimizing peptide loss and improving overall yield. This approach opens the door to the industrial-scale production of antimicrobial peptides (AMPs), as well as therapeutic, cosmetic and other peptides, in a cost-effective manner. The method is rapid, economical, and requires minimal use of chromatography—limited primarily to quality control assessments—making it highly suitable for scalable applications.

Although filtration has proven to be a highly efficient method for purifying peptides, it becomes challenging when dealing with larger proteins of similar size to the fused ELP. For larger proteins, a several-fold dilution followed by a single round of Inverse Transition Cycling can remove most of the free and fused ELP. Subsequent purification of the target protein may then be achieved using appropriate chromatographic techniques, selected based on protein’s physicochemical properties, such as size and charge.

The key advantages of this construct include:

- The ELP construct has a Tt of 27–31°C
- It requires only 200-500mM NaCl for the best outcome
- The ELP construct is fairly resistance to Tt changes upon fusion of the target protein especially with smaller peptides
- The construct can handle protein/peptides up to 45Kda
- It allows an efficient enzymatic cleavage by TEV enzyme
- The protein expression is often very high
- The purity of the final product is above 90%
- The purification is simple, cost effective and fast
- It is scalable

## Supporting information

S1 Protocol Step by step protocol for expression and purification of hBD1 peptide using a pre-configured ELP expression cassette. (PDF)

S1 File Vector sequence of ELP[V96]-hBD-1 pET-28. The full-length sequence of the vector harboring pre-configured ELP expression cassette.
(DNA)
