# Supplementary material for: Streamlining recombinant peptide and protein production using a pre-configured elastin-like polypeptide expression cassette
Source: PLoS One. 2026 Sep 11;21(9):e0357387. doi: 10.1371/journal.pone.0357387 (PMC13568509; doi:10.1371/journal.pone.0357387)
Supplement: Supplementary file 1 [file pone.0357387.s001.pdf]

Aug 26, 2026

Step by step protocol for expression and purification of human beta defensin-1 (hBD-1) peptide using a pre-configured Elastin-Like Polypeptides (ELP) expression cassette

DOI
https://dx.doi.org/10.17504/protocols.io.bp2l6ob9klqe/v1

Jetson Lobos[1], Fariborz Nasertorabi[1]

[1]Bridge Institute, Michelson Center for Convergent Bioscience, Structural Biology Center, University of Southern California, Los Angeles, CA 90089, USA

Fariborz Nasertorabi
University of Southern California

Create & collaborate more with a free account

Edit and publish protocols, collaborate in communities, share insights through comments, and track progress with run records.

Create free account

OPEN ACCESS

DOI: https://dx.doi.org/10.17504/protocols.io.bp2l6ob9klqe/v1

Protocol Citation:
Jetson Lobos, Fariborz Nasertorabi 2026. Step by step protocol for expression and purification of human beta defensin-1 (hBD-1) peptide using a pre-configured Elastin-Like Polypeptides (ELP) expression cassette. **protocols.io** https://dx.doi.org/10.17504/protocols.io.bp2l6ob9klqe/v1

License: This is an open access protocol distributed under the terms of the **Creative Commons Attribution License**, which permits unrestricted use, distribution, and reproduction in any medium, provided the original author and source are credited

Protocol status: Working
We use this protocol and it's working

Created: June 23, 2026

Last Modified: August 26, 2026

Protocol Integer ID: 319800

Disclaimer

DISCLAIMER – FOR INFORMATIONAL PURPOSES ONLY; USE AT YOUR OWN RISK

The protocol content here is for informational purposes only and does not constitute legal, medical, clinical, or safety advice, or otherwise; content added to **protocols.io** is not peer reviewed and may not have undergone a formal approval of any kind. Information presented in this protocol should not substitute for independent professional judgment, advice, diagnosis, or treatment. Any action you take or refrain from taking using or relying upon the information presented here is strictly at your own risk. You agree that neither the Company nor any of the authors, contributors, administrators, or anyone else associated with **protocols.io**, can be held responsible for your use of the information contained in or linked to this protocol or any of our Sites/Apps and Services.

Abstract

The expression and purification of hBD-1 were performed using a pre-configured ELP expression cassette, demonstrating a strategy for efficient peptide production with little to no dependence on chromatographic purification. While this approach is most effective for small peptides, larger proteins may also be purified successfully with suitable adaptations to the overall purification workflow.

Protocol materials

- kanamycin
- Terrific Broth (TB)
- AEBSF
- DNERASE, **c-LEcta**
- Lysis buffer (50mM HEPES 7.0-9.0, 200mM NaCl)
- NaCl
- IPTG
- LB (Luria–Bertani) medium
- vector expressing ELP[V-96]-hBD-1
- TEV protease

Cell Growth

14h 25m

1 Inoculate 60 mL of LB (Luria–Bertani) medium supplemented with [M] 50 µg/ml kanamycin using a single colony of expression cells harboring the vector expressing ELP[V-96]-hBD-1 . Incubate the culture Overnight at 37 °C with shaking at 220 rpm .

2 Inoculate six 2.8 L flasks, each containing 300 mL of Terrific Broth (TB) supplemented with [M] 50 µg/ml kanamycin , with 3 mL of Overnight culture per flask.

3 Let it grow at 37 °C with shaking at 220 rpm until OD reaches 0.5.

4 Reduce the temperature to 18 °C and supply the cell culture with [M] 0.25 millimolar (mM) of IPTG to induce the protein expression. Let it shake at 220 rpm for 14:00:00 at 18 °C . 14h

5 Collect the cells by centrifugation at 7000 x g, 4°C, 00:25:00 and store the cell pellet at -80 °C for future use. 25m

Purification

4h 38m 9s

6 Dissolve 20 g of dry cell paste in Lysis buffer (50mM HEPES 7.0-9.0, 200mM NaCl) in a ratio of 1 g cell paste/ 10 mL (5-10mL) lysis buffer supplied with [M] 1 millimolar (mM) AEBSF and 2500 Units (U) of DNERASE, **c-LEcta**

7 The dispersed cells are subjected to three rounds of sonication (Time: 00:03:00 , Pulse: 00:00:03 , pause: 00:00:06 , Amplitude 85%) with a ½" standard probe in an ice bath. 3m 9s

Equipment

Q500 Sonicator	NAME
QSONICA, USA	BRAND
½" standard probe	SPECIFICATIONS

8 Keep the lysed cells in an ice-cold water bath for 00:15:00 , then centrifuge at 50000 x g, 4°C, 00:50:00 .

1h 5m

9 Discard the pellet and incubate the supernatant in a water bath at Room temperature for 00:10:00 , followed by incubation at 31 °C for an additional 00:10:00 . The supernatant should become turbid by this stage.

20m

10 Add up to [M] 0.3 Molarity (M) NaCl to the supernatant to increase the aggregation, if needed.

11 **Hot Spin:** Centrifuge the supernatant at 7500 rpm, 26°C, 00:20:00 .

20m

12 Discard the sup and dissolve the pellet in 40 mL of cold Lysis buffer (50mM HEPES 7.0-9.0, 200mM NaCl) by pipetting up and down and using a glass tissue grinder.

Note

To effectively dissolve large, dense pellets, add several milliliters of the desired buffer at room temperature and initially disrupt the pellet using a wide-bore pipette tip. Transfer the suspension to a glass tissue grinder and add additional cold buffer. As a general guideline, dissolve approximately 1 g of pellet in 40 mL of final buffer solution. Continue grinding until the pellet is completely dissolved and no visible particulates remain.

13 Incubate the solution on a rotating wheel for 00:30:00 at 4 °C . 30m

14 **Cold Spin:** Centrifuge the sample at 50000 x g, 4°C, 00:20:00 . Discard the pellet and filter the supernatant with 0.45µM syringe filter. 20m

15 Add 500 µg of TEV protease to the solution to cleave and release the peptide from ELP[V-96]. Incubate the mixture on a rocking platform at Room temperature for 02:00:00 , then transfer it to 4 °C and continue the incubation Overnight . 2h

16 Load the cleaved sample onto a 15 mL centrifugal filter unit to remove the cleaved elastin-like polypeptide (ELP), any un-cleaved fusion protein, and TEV protease.

Equipment	
Centrifugal filter unit	NAME
Sigma Millipore	BRAND
UFC9030	SKU
with a 30 kDa molecular weight cut-off	SPECIFICATIONS

17 Load the filtrate obtained from the previous step onto a 15 mL centrifugal filter unit to remove any remaining TEV protease and ELP fragments. The final filtrate is collected for downstream analysis.

Equipment

**centrifugal filter unit**	NAME
Sigma Millipore	BRAND
UFC9010	SKU
with a 10 kDa molecular weight cut-off	SPECIFICATIONS

Note

If the peptide passes through 30kDa filter but cannot pass through 10kDa filter, the concentrated sample can be loaded on a size exclusion column for separation.

18 The filtrate obtained from the previous step is loaded onto a 15 mL centrifugal filter unit to concentrate the peptide.

Equipment

**centrifugal filter unit**	NAME
Pall corp	BRAND
MAP003C37	SKU
with a 3 kDa molecular weight cut-off	SPECIFICATIONS

Note

To exchange the buffer, either dilute the sample and reconcentrate it by centrifugation or load it onto a size-exclusion column.
